# Peginterferon Lambda-1a for treatment of outpatients with uncomplicated COVID-19: a randomized placebo-controlled trial

**DOI:** 10.1038/s41467-021-22177-1

**Published:** 2021-03-30

**Authors:** Prasanna Jagannathan, Jason R. Andrews, Hector Bonilla, Haley Hedlin, Karen B. Jacobson, Vidhya Balasubramanian, Natasha Purington, Savita Kamble, Christiaan R. de Vries, Orlando Quintero, Kent Feng, Catherine Ley, Dean Winslow, Jennifer Newberry, Karlie Edwards, Colin Hislop, Ingrid Choong, Yvonne Maldonado, Jeffrey Glenn, Ami Bhatt, Catherine Blish, Taia Wang, Chaitan Khosla, Benjamin A. Pinsky, Manisha Desai, Julie Parsonnet, Upinder Singh

**Affiliations:** 1 Department of Medicine, Stanford University, Stanford, CA USA; 2 Department of Microbiology and Immunology, Stanford University, Stanford, CA USA; 3 Quantitative Sciences Unit, Stanford University, Stanford, CA USA; 4 Stanford Center for Clinical Research, Stanford University, Stanford, CA USA; 5 Department of Emergency Medicine, Stanford University, Stanford, CA USA; 6 Department of Pediatrics, Stanford University, Stanford, CA USA; 7 Eiger BioPharmaceuticals, Palo Alto, CA USA; 8 Department of Genetics, Stanford University, Stanford, CA USA; 9 ChEM-H, Stanford University, Stanford, CA USA; 10 Department of Pathology, Stanford University, Stanford, CA USA

**Keywords:** Viral infection, Randomized controlled trials

## Abstract

Type III interferons have been touted as promising therapeutics in outpatients with coronavirus disease 2019 (COVID-19). We conducted a randomized, single-blind, placebo-controlled trial (NCT04331899) in 120 outpatients with mild to moderate COVID-19 to determine whether a single, 180 mcg subcutaneous dose of Peginterferon Lambda-1a (Lambda) within 72 hours of diagnosis could shorten the duration of viral shedding (primary endpoint) or symptoms (secondary endpoint). In both the 60 patients receiving Lambda and 60 receiving placebo, the median time to cessation of viral shedding was 7 days (hazard ratio [HR] = 0.81; 95% confidence interval [CI] 0.56 to 1.19). Symptoms resolved in 8 and 9 days in Lambda and placebo, respectively, and symptom duration did not differ significantly between groups (HR 0.94; 95% CI 0.64 to 1.39). Both Lambda and placebo were well-tolerated, though liver transaminase elevations were more common in the Lambda vs. placebo arm (15/60 vs 5/60; p = 0.027). In this study, a single dose of subcutaneous Peginterferon Lambda-1a neither shortened the duration of SARS-CoV-2 viral shedding nor improved symptoms in outpatients with uncomplicated COVID-19.

## Abstract (web summary)

Here the authors report the results of randomized, single-blind, placebocontrolled trial on the effects of a asingle subcutaneous dose of Peginterferon Lambda-1a (Lambda) in 120 outpatients with mild to moderate COVID-19, showing that while treatment is well tolerated it does not shorten the duration of SARS-CoV-2 viral shedding nor improves symptoms.

## Introduction

Coronavirus Disease 2019 (COVID-19), caused by the Severe Acute Respiratory Syndrome Coronavirus 2 (SARS-CoV-2), has led to more than 2 million deaths worldwide as of March 2021^1^. Although most infected patients display mild symptoms, even uncomplicated infections can contribute to transmission to those with co-morbid conditions and other high risk groups, increasing overall mortality^2^. With the unprecedented health and economic threats imposed by COVID-19, therapeutics are urgently needed to shorten the duration of viral shedding, relieve symptoms, and prevent hospitalizations.

Interferons (IFNs) are promising anti-SARS-CoV-2 therapeutics, given their importance in the early response to viral infections^3^. Innate immune sensing of viral nucleic acids leads to production of type I (IFN-α, IFN-β) and type III (IFN-λ) IFNs that, after binding to their cognate receptors, activate genes critical for host protection^4–6^. SARS-CoV-2 encodes proteins that suppress production of endogenous IFN^7^, and infection has been associated with markedly reduced type I and type III IFN signaling^8^, particularly in patients with severe manifestations of disease^9,10^. Both type I and type III IFNs inhibit SARS-CoV-2 in vitro^11,12^, suggesting potential utility of exogenous IFN administration to aid in viral control and prevent disease progression. In support of this hypothesis, recent trials in hospitalized COVID-19 patients have reported that both subcutaneous and inhaled type I IFN administration may reduce the duration of viral shedding and symptoms^13–17^.

Whereas cognate receptors to type I IFNs are expressed ubiquitously, the receptor complex (IL28R) for IFN-λ is expressed on only a few cell types, including epithelial cells in the gastrointestinal and respiratory tracts^4,18,19^. These cellular affinities have led investigators to use this agent to target viral hepatitides^20,21^ and respiratory viral infections^18^. In a murine model of influenza infection, IFN-λ treatment post-infection was associated with significantly lower mortality compared to mice treated with IFN-α, and this was associated with lower influenza viral loads^22^. A pegylated form of recombinant IFN-λ, Peginterferon Lambda-1a (Lambda) has been developed for the treatment of chronic viral hepatitis. Lambda, given weekly as 180 mcg subcutaneous injections, has comparable antiviral efficacy and an improved tolerability profile compared with type I IFN for the treatment of hepatitis^23^, likely due to its relatively limited receptor distribution. In a murine model of SARS-CoV-2 infection, subcutaneous administration of Lambda prophylactically or early after infection diminished SARS-CoV-2 replication in the lower respiratory tracts of mice in vivo^11^.

Lambda has thus emerged as a promising treatment candidate for SARS-CoV-2^24,25^ given a plausible mechanism of action, the suppression of IFN activity by SARS-CoV-2, and in vitro and in vivo studies showing that IFN-λ administration can inhibit SARS-CoV-2 replication. To date, no therapies have been approved for outpatients with mild to moderate COVID-19 disease. We therefore conducted a randomized, placebo-controlled trial of Lambda for outpatients with uncomplicated SARS-CoV-2 infection. We tested the hypothesis that a single, 180 mcg subcutaneous injection of Lambda would be associated with a shorter duration of viral shedding in comparison to a normal saline placebo injection.

In this work, we show that a single subcutaneous injection of Lambda in outpatients with uncomplicated SARS-CoV-2 infection did not significantly reduce time to viral clearance or resolution of symptoms compared with placebo. Lambda was well-tolerated, with few adverse effects, though asymptomatic liver transaminase elevations occurred more frequently in participants randomized to Lambda compared with placebo.

## Results

**Fig. 1:**
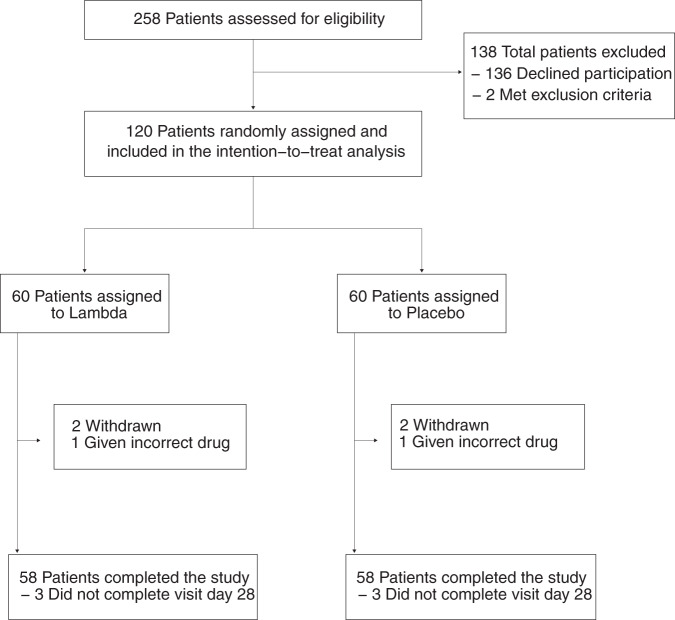
CONSORT diagram. Trial schematic showing participants screened, randomized, and followed through study completion.

We enrolled 120 participants between April 25 and July 17, 2020, of whom 110 (91.7%) completed 28 days of follow up (Fig. 1a). The median age was 36 years (range 18–71), 50 participants (41.7%) were female, and 75 (62.5%) were Latinx ethnicity (Table 1). Eight (6.7%) participants were asymptomatic at baseline. Of those with symptoms, the median duration of symptoms prior to randomization was 5 days. The most common symptoms were fatigue, cough, headache, and myalgias (Table 1, Supplementary Fig. 1). Only 13 (10.8%) participants had an elevated temperature (>99.5°F) at baseline; the median oxygen saturation was 98%. The proportion of missing follow-up visits was 44/960 (4.6%). Only 16/960 visits were missed among patients not hospitalized or prematurely withdrawn.

**Table 1:** Baseline characteristics of study participants.

	Treatment arm		
	Lambda (*n* = 60)	Placebo (*n* = 60)	Overall (*n* = 120)
Age in years, median (range)	37 (18–66)	34 (20–71)	36 (18–71)
Female, *n* (%)	24 (40.0%)	26 (43.3%)	50 (41.7%)
Race/Ethnicity, *n* (%)			
Latinx	34 (56.7%)	41 (68.3%)	75 (62.5%)
White	18 (30.0%)	15 (25.0%)	33 (27.5%)
Asian	3 (5.0%)	4 (6.7%)	7 (5.8%)
Native Hawaiian or other Pacific Islander	2 (3.3%)	0 (0%)	2 (1.7%)
Unknown	2 (3.3%)	0 (0%)	2 (1.7%)
More than one race	1 (1.7%)	0 (0%)	1 (0.8%)
BMI (kg/m^2^), median (IQR)	27.6 (25.4–31.1)	28.5 (24.8–32.3)	27.7 (24.9–32.0)
Comorbid conditions			
Hypertension	9 (15.0)	5 (8.3)	14 (11.7)
Diabetes	4 (6.7)	8 (13.3)	12 (10.0)
Asthma	2 (3.3)	2 (3.3)	4 (3.3)
Heart disease	3 (5.0)	1 (1.7)	4 (3.3)
Concomitant medications			
Antihypertensive	9 (15.0)	5 (8.3)	14 (11.7)
Oral hypoglycemic	4 (6.7)	8 (13.3)	12 (10.0)
Insulin	0 (0.0)	1 (1.7)	1 (0.8)
Aspirin	1 (1.7)	2 (3.3)	3 (2.5)
Statin	3 (5.0)	5 (8.3)	8 (6.7)
Albuterol	2 (3.3)	0 (0.0)	2 (1.7)
Asymptomatic at baseline, *n* (%)	6 (10.0%)	2 (3.3%)	8 (6.7%)
Duration of symptoms in days prior to randomization, median (IQR) ^a^	4 (3–6)	5 (3–5)	5 (3–6)
Symptoms at baseline, *n* (%)			
Fatigue	33 (55%)	42 (70%)	75 (62.5%)
Cough	33 (55.0%)	36 (60.0%)	69 (57.5%)
Headache	29 (48.3%)	36 (60%)	65 (54.2%)
Myalgias	29 (48.3%)	34 (56.7%)	63 (52.5%)
Decreased taste or smell	25 (41.7%)	32 (53.3%)	57 (47.5%)
Chills	22 (36.7%)	27 (45%)	49 (40.8%)
Sore throat	22 (36.7%)	23 (38.3%)	45 (37.5%)
Joint pain	19 (31.7%)	19 (31.7%)	38 (31.7%)
Diarrhea	16 (26.7%)	18 (30%)	34 (28.3%)
Nausea	11 (18.3%)	23 (38.3%)	34 (28.3%)
Shortness of breath	16 (26.7%)	16 (26.7%)	32 (26.7%)
Chest pain/pressure	14 (23.3%)	13 (21.7%)	27 (22.5%)
Runny nose	10 (16.7%)	16 (26.7%)	26 (21.7%)
Abdominal pain	7 (11.7%)	7 (11.7%)	14 (11.7%)
Rash	4 (6.7%)	5 (8.3%)	9 (7.5%)
Vomiting	1 (1.7%)	5 (8.3%)	6 (5%)
Vital signs at enrollment			
Temperature 99.5F+, n (%)	6 (10.0%)	7 (11.7%)	13 (10.8%)
Oxygen saturation, median (IQR)	98 (2.5)	99 (3)	98 (3)
Baseline laboratory values, median (IQR)			
White blood cell (WBC) count, cells/µl	5.5 (4.3–6.8)	5.6 (4.0–7.5)	5.5 (4.1–7.1)
Absolute lymphocyte count (ALC), cells/µl	1.5 (1.2–1.9)	1.5 (1.2–2.3)	1.5 (1.2–2.2)
Aspartate aminotransferase, IU/L	31 (26–41)	30 (25–39.3)	30 (25–41)
Alanine aminotransferase, IU/L	32.5 (21–52.3)	30.5 (23–47.5)	31.5 (22–50.3)
Baseline oropharyngeal SARS-CoV-2 cycle threshold, median (IQR)^b^	30.9 (26.4–33.8)	29.3 (26.4–34.3)	30.3 (26.4–34.3)
Baseline Log10 Viral Load, median (IQR)^b^	4.2 (3.3–5.5)	4.7 (3.2–5.5)	4.4 (3.2–5.5)
Baseline SARS-CoV-2 IgG seropositivity, *n* (%)	21 (35.0%)	28 (46.7%)	49 (40.8%)
Sum of risk factors, median (IQR)	3 (2–3)	3 (2–4)	3 (2–3)

Sum of risk factors is defined as the number of relevant severe disease risk factors present at baseline (presence of either temperature of 99.5F+, cough, or shortness of breath; age 60+; male sex; Black race; Latinx ethnicity; BMI 30+; ALC < 1000; ALT 94+). Source data available at https://purl.stanford.edu/hc972ys6733.

*IQR* inner quartile range, *ASD* absolute standardized difference.

^a^Among *n* = 103 participant who reported symptoms prior to randomization (*n* = 48 in lambda and *n* = 55 in placebo).

^b^Among *n* = 87 participants with detectable OP virus (*n* = 44 in lambda and *n* = 43 in placebo).

The presence of SARS-CoV-2 RNA was assessed at baseline and at 8 follow-up visits using oropharyngeal swabs, a Centers for Disease Control approved method for SARS-CoV-2 detection^26^. This method was selected given the frequency of repeated assessments and improved tolerability for participants compared with nasopharyngeal swabs. The median SARS-CoV-2 oropharyngeal viral PCR cycle threshold at enrollment was 30.3 (corresponding to a median viral load of 4.4 Log10 copies/ml), and was comparable between groups (Table 1). Patients were randomized within a median of 2 days (range 0–3 days) following diagnosis. The median time from onset of symptoms before diagnosis was 5 days (IQR 3–6).

IgG antibody titers against the SARS-CoV-2 spike receptor-binding domain (RBD) were assessed at enrollment by enzyme-linked immunosorbent assay^27^. At enrollment, 49 (40.8%) participants were SARS-CoV-2 IgG seropositive. Baseline SARS-CoV-2 serostatus did not significantly differ between groups (Table 1). The median duration of symptoms prior to enrollment was shorter in SARS-CoV-2 IgG seronegative vs. seropositive participants (median [interquartile range [IQR]]: 3.5 [2.5–5] days vs. 5 [4–7] days, *P* = 0.0051). Seronegative participants also had significantly higher oropharyngeal viral RNA levels at enrollment compared with seropositive participants (median [IQR] log_10_ viral load 4.4 [2.5] vs. 2.0 [2.4]).

**Fig. 2:**
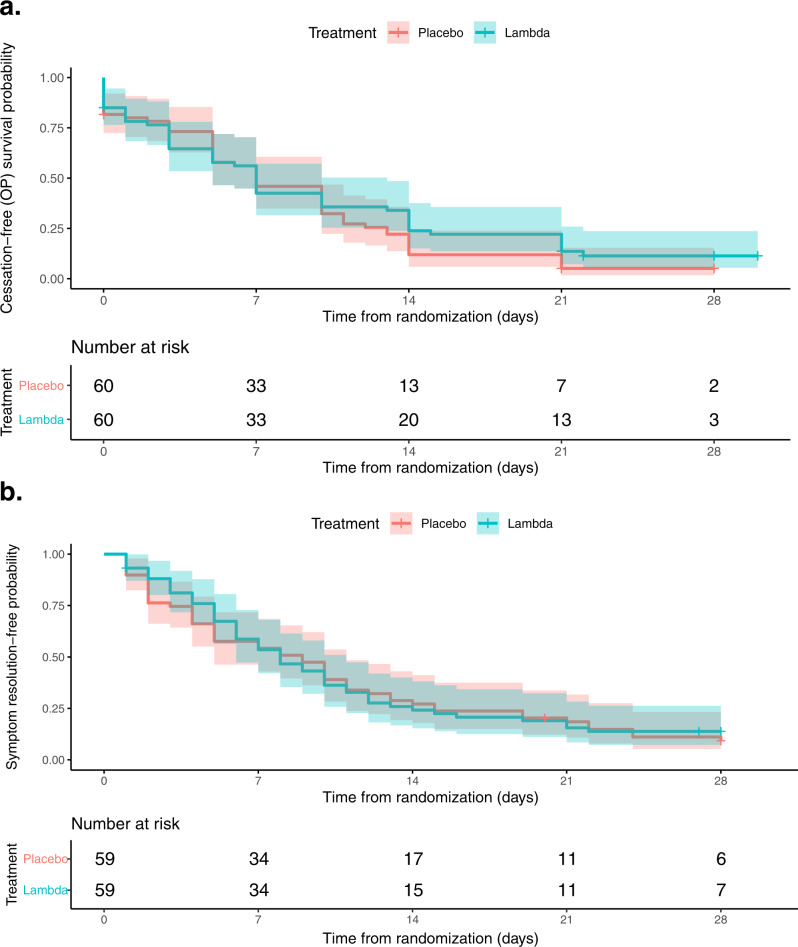
Kaplan–Meier analyses of the primary and key secondary outcome in the intention-to-treat population. **a** Time until cessation of SARS-CoV-2 viral shedding from oropharyngeal swabs stratified by treatment arm, Lambda (blue) vs. placebo (red). **b** Time until resolution of all symptoms stratified by treatment arm, Lambda (blue) vs. placebo (red). In both panels, solid lines represent Kaplan–Meier survival probability; shading represents 95% confidence intervals. Source data available at https://purl.stanford.edu/hc972ys6733.

Of 120 enrolled participants, 60 were randomized to receive Lambda and 60 randomized to receive placebo and included in the analysis. The median time to cessation of oropharyngeal viral shedding was 7 days in both arms (95% CI 5–10 days for placebo vs. 5–13 days for Lambda, Table 2). There was no significant difference in the adjusted hazard ratio (aHR) for shedding cessation between Lambda and placebo; participants in the Lambda arm were 19% less likely to cease shedding at any point during the study period compared to participants in the placebo arm (aHR 0.81, 95% confidence interval [CI] 0.56– 1.19; *p* = 0.29, Fig. 2a). Overall, 108 participants met the primary endpoint and were not censored. Because two participants, after randomization, inadvertently were injected with the incorrect syringe, we also conducted an as-treated analysis according to treatment actually received. Findings from an as-treated analysis (aHR 0.83, 95% CI 0.56–1.21; *p* = 0.33) and an analysis performed in symptomatic patients only (aHR 0.77, 95% CI 0.52–1.15, *p* = 0.21) were similar.

**Table 2:** Efficacy and safety outcomes.

	Treatment arm		Measure of association	
	Lambda (*n* = 60)	Placebo (*n* = 60)	aHR (95% CI)	*p*-value
*Primary efficacy outcome*				
Duration until viral shedding cessation in days, median (95% CI)	7 (5–13)	7 (5–10)	0.81 (0.56, 1.19)	0.29
*Secondary efficacy outcomes*				
Duration until resolution of symptoms in days, median (95% CI)	8 (6–11)	9 (5–11)	0.94 (0.64, 1.39)	0.76
Hospitalizations by Day 28, *n* participants (%)	2 (3.3%)	2 (3.3%)	–	1
Emergency Department visits by Day 28, *n* participants (%)	5 (8.3%)	3 (5%)	–	0.71
			**∆ Log change (95% CI)**	***p*-value**
Log Oropharyngeal viral load over time, mean change at day 14 (SD)	−4.3 (4.3)	−4.9 (4.7)	−0.06 (−1.23, 1.11)	0.91
			**∆ AUC (95% CI)**	***p*-value**
Log_10_ viral load area under the curve through day 14, median (IQR)	28.5 (20.1)	29.6 (19.0)	1.01 (0.85, 1.16)	0.95
*Exploratory efficacy outcomes*			**aHR (95% CI)**	***p*-value**
Duration until sustained symptom resolution in days, median (95% CI)	20 (16–27)	20 (17–24)	0.92 (0.60, 1.41)	0.70
Duration until respiratory symptom resolution in days, median (95% CI)	6 (4–7)	4 (2–7)	0.99 (0.64, 1.53)	0.95
Duration until systemic and respiratory symptom resolution in days, median (95% CI)	8 (6–11)	5.5 (5–10)	0.93 (0.63, 1.38)	0.73
Duration until disease progression in days, median among those who progress* (IQR)	5 (1)	2 (1)	1.38 (0.52, 3.63)	0.52
*Safety outcomes*				
Serious adverse events, *n* (%)	2 (3.3%)	2 (3.3%)	–	1
Number of adverse events, *n*	36	30	–	
Participants with adverse events, *n* (%)	25 (41.7%)	21 (35.0%)	–	0.57
LFT-related adverse events, *n*	16	5	–	
Participants with LFT-related adverse events, *n* (%)	15 (25.0%)	5 (8.3%)	–	0.027

Source data available at https://purl.stanford.edu/hc972ys6733.

*IQR* inner quartile range; *aHR* adjusted hazard ratio (adjusted for age 50+ and sex), aHR > 1 favors faster shedding cessation in Lambda vs. placebo arms; aHR <1 favors delayed shedding cessation in Lambda vs. placebo arms, *AUC* area under the curve.

^a^Number of participants that progressed—10 in Lambda, 7 in placebo.

No significant difference in time to resolution of symptoms (aHR 0.94; 95% CI 0.64–1.39; *p* = 0.76, Fig. 2b) or sustained resolution of symptoms (aHR 0.92; 95% CI 0.60–1.41; *p* = 0.70) was observed, nor did we find any significant difference in resolution of symptom complexes (Table 2, Supplementary Fig. 2). Overall, 17 participants had evidence of disease progression, defined as hospitalization, presentation to the emergency department, or worsening cough or shortness of breath defined as an increase in severity of two points or more on a five-point scale. Time to clinical progression was not significantly different between the two arms (aHR 1.38; 95% CI 0.52–3.63; *p* = 0.52, Table 2). At-home oxygen saturation measurements also did not differ significantly between groups (Supplementary Fig. 3).

**Fig. 3:**
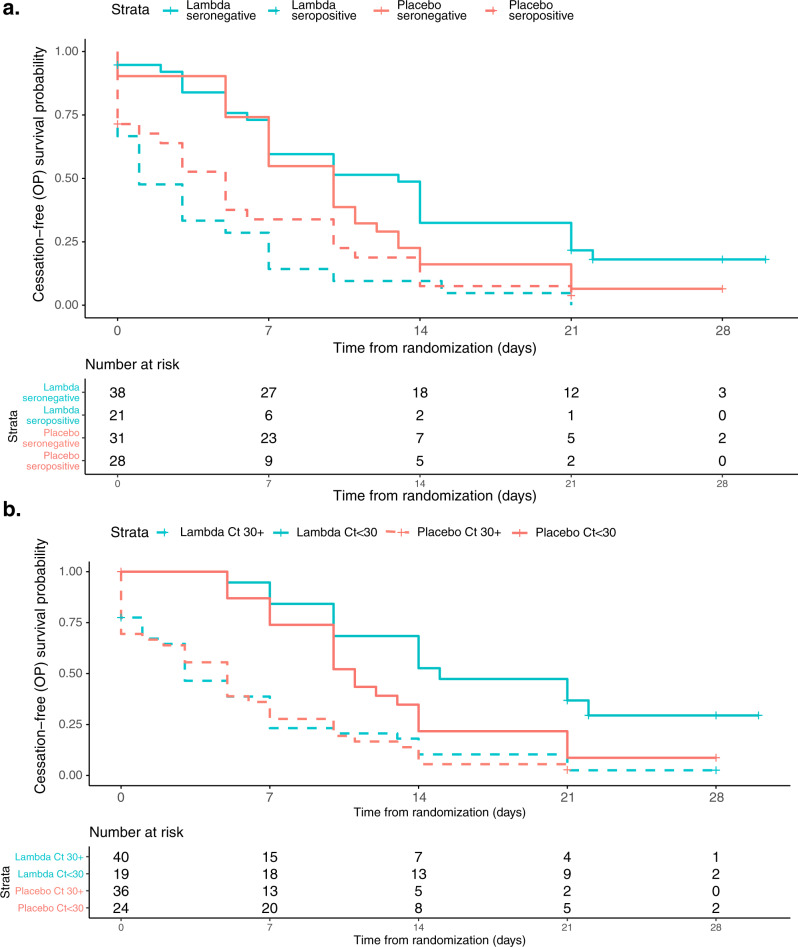
Kaplan–Meier analyses of the primary outcome, stratified by baseline seropositivity and viral load. **a** Time until cessation of SARS-CoV-2 viral shedding from oropharyngeal swabs stratified by baseline SARS-CoV-2 seropositivity, seropositive (dashed) and seronegative (solid), and treatment arm, Lambda (blue) vs. placebo (red). **b** Time until cessation of SARS-CoV-2 viral shedding from oropharyngeal swabs stratified by baseline SARS-CoV-2 oropharyngeal virus CT value, CT value ≥ 30 (dashed) and CT value < 30 (solid), and treatment arm, Lambda (blue) vs. placebo (red). In both panels, solid lines represent Kaplan–Meier survival probability; shading represents 95% confidence intervals. Source data available at https://purl.stanford.edu/hc972ys6733.

Trajectory of viral RNA levels did not vary by treatment arm (*p* = 0.91, Supplementary Fig. 4) nor did viral RNA area under the curve (*p* = 0.95, Table 2). In exploratory analysis, seropositivity at baseline was associated with significantly hastened shedding cessation. The median time to shedding cessation was 10 days in SARS-CoV-2 IgG seronegative individuals vs. 3 days in seropositive individuals (95% CI 7–14 days for seronegative vs. 1–6 days for seropositive, aHR 2.65, 95% CI 1.74–4.03, p < 0.001, Supplementary Fig. 5). Baseline serostatus significantly modified the effect of treatment on time to shedding cessation (*p* = 0.03). Among seronegative individuals, point estimates suggest that Lambda delayed shedding cessation compared with placebo (aHR 0.66, 95% CI 0.39–1.10, Fig. 3a). Among seropositive individuals, point estimates suggest that Lambda hastened shedding cessation compared with placebo (aHR 1.58, 95% CI 0.88–2.86, Fig. 3a).

Higher oropharyngeal viral RNA levels at baseline were associated with significantly delayed shedding cessation (aHR 0.32 comparing baseline CT <30 vs. baseline CT ≥ 30, 95% CI 0.21–0.50, *p* < 0.001, Supplementary Fig. 6). Although baseline oropharyngeal viral RNA levels did not significantly modify the effect of treatment on time to shedding cessation (*p* = 0.15), among individuals with high baseline viral RNA levels, Lambda tended to delay shedding cessation compared with placebo (aHR 0.51, 95% CI 0.26–1.04, Fig. 3b). There was no difference in shedding cessation between arms with low baseline viral RNA levels (aHR 0.95, 95% CI 0.60–1.52, Fig. 3b). No other baseline features of interest significantly modified the effect of treatment and the primary outcome (Table S1).

Twenty-five (42%) Lambda and 21 (35%) placebo participants experienced adverse events (Table 2). Two serious adverse events (hospitalizations) were reported in each arm, with all four hospitalizations due to hypoxia/worsening COVID-19. Liver transaminase elevations were more common in the Lambda vs. placebo arm (15 vs. 5; *p* = 0.027). Furthermore, we observed significant elevations in alanine transaminase levels from day 0 to day 5 among individuals randomized to Lambda, but not among individuals randomized to placebo (Supplementary Fig. 7). However, there were no associated symptoms and lab abnormalities were not sustained.

## Discussion

In this study of outpatients with uncomplicated SARS-CoV-2 infection, a single subcutaneous injection of Lambda did not significantly reduce time to viral clearance or resolution of symptoms compared with placebo. We recruited participants within 72 h of diagnosis, giving us an excellent opportunity to intervene early within the course of infection. We attained excellent follow-up and retention, with few missed visits (<5%), and little missing data. Despite these strengths and compelling preclinical data—i.e., a plausible mechanism of action, the suppression of IFN activity by respiratory coronaviruses, and both in vitro and in vivo studies showing inhibition of SARS-CoV-2 replication by IFN-λ^11,12^—this phase 2 trial yielded little promise of efficacy at the tested dose and administration schedule. Lambda was well-tolerated, with few adverse effects, though asymptomatic liver transaminase elevations occurred more frequently in participants randomized to Lambda and are consistent with previous reports^23,28^.

The lack of effect of Lambda was surprising given recently described in vitro data and benefits seen in an in vivo model with early therapeutic and prophylactic administration^12^. There are several potential reasons for this lack of benefit. First, a single, 180 μg subcutaneous injection of Lambda may not achieve adequate therapeutic levels of drug in the upper respiratory epithelia. In an in vitro model of SARS-CoV-2 infection, human epithelial cells pre-treated with 10 ng/ml of Lambda had a ~1 log reduction in viral titers. It is unclear whether these tissue levels are achievable, since, in Phase 1 and Phase 2 studies of single-dose subcutaneous administration of 180 μg Lambda, *C*_max_ values ranged from 1.06 to 2.41 ng/mL (median *T*_max_, 8–25 h, range 1–120 h^29^.) Consistent with this, a murine model of SARS-CoV-2 infection found that subcutaneous administration of Lambda resulted in significant reductions of SARS-CoV-2 viral titers in lower, but not upper, respiratory epithelium^12^. The dose of Lambda used in this model was 2 μg per mouse; the human equivalent dose (HED) is 8.1 μg/kg/dose^30^. In an average 70 kg adult, 180 μg Lambda would result in 2.6 μg/kg/dose, 1/3 of the dose given to mice. Though higher, or more frequent, dosing may have been beneficial, subcutaneous doses >180 mcg in humans are limited by increasing drug toxicity, including significant liver transaminase elevations^23^. Second, although we attempted to randomize participants as soon as possible after the COVID-19 diagnosis was made, the median symptom duration was 5 days at the time of randomization, and 40% of participants were already SARS-CoV-2 IgG positive at enrollment. It is possible that earlier administration, or prophylactic administration prior to established infection, would have been beneficial. Arguing against this, we observed no evidence of benefit among SARS-CoV-2 seronegative individuals, who presumably have been infected a shorter period of time. Finally, IFN-λ has been shown to disrupt the lung epithelial barrier in mice, leading to worsened disease course and increase susceptibility to bacterial superinfection^31,32^. Furthermore, IFN-λ has also been shown to inhibit influenza virus-stimulated B-cell activation and antibody production^33^, suggesting negative impacts on the development of adaptive immune responses critical to resolution of infection. These may negate any positive antiviral effects.

Our results differ somewhat from those seen in a similar, but smaller, randomized controlled trial of Lambda at the same dose in COVID-19 outpatients in Toronto^34^. In the Toronto study, Feld and colleagues randomized 60 outpatients to a single subcutaneous injection of 180 μg Lambda (*n* = 30) or placebo (*n* = 30) within 7 days of symptom onset or first swab if asymptomatic. The primary outcome was proportion negative for SARS-CoV-2 RNA by self-collected mid turbinate swabs on day 7 post injection. The authors found that, overall, 24/30 (80%) individuals in the Lambda arm, vs. 19/30 (63%) in the placebo arm, were negative for SARS-CoV-2 RNA on day 7 (*p* = 0.15). However, baseline mid-turbinate viral loads were higher in the Lambda arm (6.16 log10 copies/ml) vs. the placebo arm (4.87 log10 copies/ml) among those with detectable viral loads at enrollment. After adjusting for baseline viral load, patients in the Lambda group were more likely to be clear of virus by Day 7 after dosing (OR 4.12 95% CI 1.2 16–7, *p* = 0.029). Among individuals with mid-turbinate viral loads of >10^6^ copies/ml at enrollment (19 in the lambda arm vs. 16 in placebo), a greater proportion of Lambda participants were negative at day 7 than those in placebo (15/19 (79%) in Lambda vs. 6/16 (38%) in placebo, *p* = 0.01); no significant difference was observed among individuals with viral loads of <10^6^ copies/ml at enrollment. While these secondary analyses adjusting for baseline viral load were pre-specified, they were not the primary outcome. Thus, given the relatively small sample size among those with higher viral loads (*n* = 35), the corresponding type I error for this particular result is unclear. In contrast to this finding, despite a similar duration of viral shedding among higher viral load subjects, in our study, Lambda appeared to prolong shedding relative to placebo among those with higher baseline viral RNA levels. Furthermore, Lambda appeared to prolong shedding relative to placebo among those who were seronegative at baseline. Although these subgroup analyses were exploratory and should only be considered hypothesis generating, our findings are not consistent with a possible benefit of Lambda among individuals with higher viral loads (or seronegative individuals early in the course of disease).

These data are in contrast to reports of benefits of Type I IFN in hospitalized COVID-19 patients. Subcutaneous IFN-β along with ribavirin and lopinavir/ritonavir was associated with shortened duration of symptoms and viral shedding in hospitalized patients in Hong Kong^14^, and a reduction in mortality when given with hydroxychloroquine and lopinavir/ritonavir or atazanavir-ritonavir in Iran^17^. Furthermore, a randomized clinical trial in England also suggest clinical benefits of inhaled IFN-β for COVID-19^15^. Although both type I and type III IFN activate the same dominant JAK-STAT signaling pathway^6^, inhibit SARS-CoV-2 in vitro^10,11^, and have receptors on respiratory epithelia^18^, in vivo activity and efficacy may differ^6^. A recent report found that inborn errors of Type I IFN immunity, including autosomal recessive IFNAR1 deficiency, were enriched in patients with life-threatening COVID-19 pneumonia^35^. Furthermore, patients with COVID-19 pneumonia were also more likely to have neutralizing auto-antibodies against type I, but not type III, IFNs^36^. These data suggest the possibility that type I IFN administration may be more beneficial than type III IFN in preventing adverse outcomes of SARS-CoV-2 infection.

The majority (62.5%) of participants in our study were Latinx, reflecting the high burden of COVID-19 among the Latinx community in our surrounding communities^37^. Minority populations are disproportionately affected by COVID-19, with higher rates of infection and deaths observed due to a multitude of socioeconomic and demographic factors^38,39^. Attention has recently been called to the relative absence of the most affected minorities in treatment trials^40,41^, and we prioritized recruitment efforts to the Latinx community in our study.

The study did have a few limitations. We recruited both symptomatic and asymptomatic patients. Asymptomatic patients contributed less to secondary outcomes since they presented with lower viral RNA levels and could not contribute to analyses of symptom alleviation. However, these patients represented <10% of the enrolled cohort. Additionally, despite a reported median duration of symptoms prior to randomization of only 5 days, 40% of participants were already seropositive at enrollment. A recently published outpatient study of a monoclonal antibody cocktail with similar study design (REGN-COV2) found similar rates of baseline SARS-CoV-2 IgG seropositivity (45%)^42^. These data suggest that enrolling COVID-19 outpatients early in the course of disease, before they develop an antibody response, may be challenging. Nonetheless, we found no suggestion of benefit of Lambda in seronegative individuals. We do not provide data on viral measures indicative of active replication (e.g., tissue culture infectious dose (TCID)_50_ assays), although samples with CT values >24 have been reported to have very low levels of being tissue culture positive^43^. Finally, the median time to cessation in the placebo arm was shorter than assumed in our sample size calculations, potentially due to less severe disease in this population. However, our original sample size estimates based on the number of events and median time to event were conservative; a shorter time to cessation, keeping all other assumptions the same, increases the power to detect differences between groups.

In conclusion, a single dose of subcutaneous Peginterferon Lambda-1a, while safe, neither reduced time to cessation of viral shedding nor symptom duration in outpatients with uncomplicated COVID-19 in this large, Phase 2, single-center study. Further investigation into the therapeutic utility of subcutaneous Peginterferon Lambda-1a for COVID-19 in patients with severe illness (NCT04343976) or as a prophylactic treatment (NCT04344600) are underway.

## Methods

### Trial design and oversight

We conducted a Phase 2, single-blind, randomized placebo-controlled trial to evaluate the efficacy of Lambda in reducing the duration of viral shedding in outpatients. The trial was conducted within the Stanford Health Care System. Adults aged 18–65 years with an FDA emergency use authorized reverse transcription-polymerase chain reaction (RT-PCR) positive for SARS-CoV-2 within 72 h from swab to the time of enrollment were eligible for participation in this study. Both symptomatic and asymptomatic patients were included given reports finding that both CT values and presence of infectious virus were similar in samples from asymptomatic and symptomatic persons^44^. Symptomatic individuals were eligible given the presence of mild to moderate symptoms without signs of respiratory distress. Asymptomatic individuals were eligible if infection was initial diagnosis of SARS-CoV-2 infection. Exclusion criteria included current or imminent hospitalization, respiratory rate >20 breaths per minute, room air oxygen saturation <94%, pregnancy or breastfeeding, history of decompensated liver disease, recent use of interferons, antibiotics, anticoagulants or other investigational and/or immunomodulatory agents for treatment of COVID-19, and prespecified lab abnormalities. Full eligibility criteria are provided in the study protocol. The protocol was amended on June 16, 2020 after 54 participants were enrolled but before results were available to include adults up to 75 years of age and eliminate exclusion criteria for low white blood cell and lymphocyte count. The trial was registered at ClinicalTrials.gov (NCT04331899). The study was performed as an investigator initiated clinical trial with the FDA (IND 419217), and approved by the Institutional Review Board of Stanford University.

### Recruitment and enrollment

Participants were recruited with flyers, online advertising, and phone calls to Stanford patients with positive SARS-CoV-2 RT-PCR. Recruitment materials and phone calls were provided in multiple languages, including English and Spanish. After confirming eligibility and providing informed consent in the patient’s primary language, participants underwent a standardized history and physical exam, and completed bloodwork. If inclusion criteria were met, participants were randomly assigned to Lambda or placebo using a 1:1 REDCAP-based computer-generated randomization scheme that stratified by age (≥50 and <50 years old) and sex. A password-protected electronic spreadsheet containing the randomization allocation, along with the code used to generate the allocation and seed used in the random number generation, was stored on secure servers at Stanford.

### Study drug administration

Phase 2 studies established the optimal dose for virologic suppression and minimizing treatment-related adverse events (mainly aminotransferase and/or bilirubin elevations) for hepatitis C at 180 mcg given subcutaneously^23^. This dose is also currently being used in hepatitis D trials, and was provided by Eiger BioPharmaceuticals for use in this study. Those assigned to Lambda received a single 180 mcg subcutaneous injection of study drug (0.45 ml volume), and those assigned to placebo received a 0.45 ml subcutaneous injection of saline (prepared by the Stanford Investigational Pharmacy). A single injection was chosen given Lambda’s prolonged half-life (mean *T*_1/2_ 63–117 h^29^) and ease of single-dose administration. The study medication/placebo syringe was dispensed by the Stanford Investigational Pharmacist and administered by a study nurse. Lambda and placebo syringes were identically labeled but differed in the appearance of the needle hub. Since the nurse administering the medication might see syringe differences, the study was not strictly “double-blind” even though all participants and investigators were blinded to treatment arm. Participants were monitored for adverse events for thirty minutes after injection.

### Participant follow up

Participants completed a daily symptom questionnaire using REDCap Cloud version 1.5. Participants also provided in-home measurements of temperature and oxygen saturation using study-provided devices. In-person follow-up visits were conducted at Day 1, 3, 5, 7, 10, 14, 21, and 28, with assessment of symptoms and vitals, and collection of oropharyngeal swabs (FLOQ Swabs; Copan Diagnostics). Peripheral blood was also collected at Day 5 and 14 to assess for safety events.

### Laboratory procedures

Laboratory measurements were performed by trained study personnel using point-of-care CLIA-waived devices or in the Stanford Health Care Clinical Laboratory. Oropharyngeal swabs were tested for SARS-CoV-2 in the Stanford Clinical Virology Laboratory using an emergency use authorized, laboratory-developed, RT-PCR^45–47^. Centers for Disease Control and Prevention guidelines identify oropharyngeal swabs as acceptable upper respiratory specimens to test for the presence of SARS-CoV-2 RNA^26^, and detection of SARS-CoV-2 RNA swabs using oropharyngeal swabs was analytically validated in the Stanford virology laboratory.

IgG antibody titers against the SARS-CoV-2 spike receptor-binding domain (RBD) were assessed at enrollment by enzyme-linked immunosorbent assay (ELISA)^27^. Briefly, His_6_-tagged SARS-CoV-2 RBD construct (gift from F. Krammer, Icahn School of Medicine at Mount Sinai) was transiently transfected into Expi293F cells (Thermo Fisher Scientific). After transfection, cell culture supernatants were collected by centrifugation for protein purification. Protein purity was assessed by SDS–PAGE. Round-bottom 96-well plates (Immunolon 2HB; Thermo Scientific) were coated with 50 μl of RBD at 2 μg ml^−1^ in PBS. Heat-inactivated serum samples at enrollment were diluted 5-fold starting at 1:50 and IgG antibody titers against RBD determined by ELISA. Absorbance was measured at 450 nm (SPECTRAmax 250, Molecular Devices). Samples were considered seropositive against RBD if their absorbance value was greater than the mean plus four standard deviation (SD) of all negative controls (*n* = 130).

### Data and safety monitoring

Adverse events were assessed and graded for severity according to standardized criteria^20^. A Data and Safety Monitoring Board (DSMB) was established and conducted an interim analysis to review clinical trial progress, integrity, and safety data.

### Study outcomes

The primary pre-specified outcome was time to first of two consecutive negative oropharyngeal tests for SARS-CoV-2 by RT-PCR. Secondary outcomes included: (1) Time to alleviation of all symptoms, defined as time until the first day when no symptoms were reported; (2) SARS-CoV-2 oropharyngeal viral RNA levels over time; (3) SARS-CoV-2 oropharyngeal viral RNA area under the curve (AUC); and (4) Incidence of emergency department visits or hospitalizations within 28 days of initiation of treatment. Adverse events (AEs) and serious AEs (SAEs) were the primary safety endpoints. For secondary outcomes utilizing oropharyngeal viral RNA levels, we used the following conversion formula from cycle threshold values to copies/ml PBS:

1 $$10 \wedge \left( { - 0.288 \ast {\mathrm{Ct}} + 11.007} \right) \times 120$$

Exploratory outcomes included: (1) time until sustained resolution of symptoms, defined as the first day when no symptoms were reported for the duration of the study; (2) Progression of disease, defined as admission to the emergency department, hospitalization, or worsening cough or shortness of breath defined as an increase in severity of two points or more on a five-point scale. Primary, secondary, and exploratory outcomes were pre-specified in our statistical analysis plan and finalized prior to database lock.

### Statistical analysis

Analyses were performed according to assigned randomization arm (intent-to-treat). All models were covariate-adjusted for the randomization strata of age group and sex. Unless otherwise noted, all tests were two-sided and conducted at the 0.05 level of significance. Analyses were performed in R version 4.0.2^48^.

#### Primary analysis

Time until shedding cessation was compared between arms using a Cox proportional hazards model covariate-adjusted for age and sex, with the final hypothesis test conducted at the alpha = 0.04999 level of significance to allow for an interim analysis. The hazard ratio for shedding cessation was estimated, along with its 95% confidence interval. Participants who dropped out prior to having two consecutive negative tests were censored at the time of their last positive test or on Day 1 if no positive tests were observed. The proportional hazards assumption was verified by examining the Schoenfeld residuals. Efron’s approximation was used to handle ties in the Cox proportional hazards model.

#### Secondary analysis

Time until resolution of symptoms was compared using a Cox proportional hazards model. Hazard ratios and 95% confidence intervals were reported. Change in viral load during follow-up was compared using a linear mixed-effects model with random intercepts for participant. The AUC of viral load was compared using linear regression. Multiple imputation using chained equations was used to impute missing viral load data prior to area under the curve calculation. Five data sets were imputed, and imputed values calculated using non-missing viral load on each of the 7 sample collection days, treatment arm, age, sex, and whether or not a participant was hospitalized. Model estimates were pooled across the five imputed datasets by computing the total variance over the repeated analyses.

Estimates for change in viral load and viral load AUC for lambda compared to placebo and corresponding 95% confidence intervals for the linear models were reported. The incidence of hospitalizations and emergency department visits was estimated for each arm, with 95% confidence intervals. AEs were compared by arm using the Chi-squared test and Fisher’s exact test for SAEs.

A sensitivity analysis was performed for the primary endpoint using only symptomatic patients at baseline. Because two participants, after randomization, inadvertently were injected with the incorrect syringe, we also conducted an as-treated analysis according to treatment actually received.

A statistical interaction term between treatment arm and symptomatic status at baseline was added to the Cox proportional hazards model adjusted for age group and sex to test whether symptomatic status was an effect modifier of the relationship between treatment and time to shedding cessation. The main effect of each potential effect modifier was also included in the model. Additional effect modifiers specified a priori were (1) having a CT value <30 (vs. ≥30) on baseline oropharyngeal swab, (2) IgG seropositivity at baseline, and (3) number of risk factors or predictors for severe disease present at baseline (temperature ≥ 99.5, cough, or shortness of breath present at randomization [symptoms count as a single risk factor], age ≥ 60, male sex, Black race, Hispanic ethnicity, body mass index ≥ 30, and lab values of baseline lymphocyte counts < 1000 and baseline ALT ≥ 94). Effect modification was considered significant if the *p* value for interaction was <0.05.

Post hoc analyses were conducted to test for differences in both median duration of symptoms pre-randomization and baseline log_10_ viral load between seronegative and seropositive participants. The Kruskal–Wallis rank sum was used to test for differences in symptom duration while a two-sample *t*-test was used to test for differences in log_10_ viral load.

### Sample size determination

Assuming 1:1 randomization and the use of a two-sided log rank test at the alpha = 0.04999 level of significance for the final analysis, we anticipated the occurrence of 79 shedding cessation events, which provided 80% power to detect a hazard ratio of 2.03. We additionally assumed median time to shedding cessation of 14 days in the control arm and 7 days in the treatment arm, a 2-month accrual period, a 2-week follow-up period after randomization of the last patient, and 10% drop out in the control arm. This enabled an interim analysis conducted at alpha = 0·00001 to assess overwhelming efficacy after 50% of participants completed 24 h of follow-up. We estimated that the total sample size required to achieve 79 events was 120 (60 participants per arm).

### Ethical approval

The study was registered as an investigator initiated clinical trial with the FDA (IND 419217), and approved by the Institutional Review Board of Stanford University. Written informed consent was provided by all study participants.

### Reporting summary

Further information on research design is available in the Nature Research Reporting Summary linked to this article.

## Supplementary information

Supplementary Information

Peer Review File

Reporting Summary

## Data Availability

The datasets generated and analyzed during the current study are permanently available in the Stanford Digital Repository at the following URL: https://purl.stanford.edu/hc972ys6733 Source data underlying all figures including those in supplementary information are provided at the above URL.
