# Supplementary material for: Peginterferon Lambda-1a for treatment of outpatients with uncomplicated COVID-19: a randomized placebo-controlled trial
Source: Nat Commun. 2021 Mar 30;12:1967. doi: 10.1038/s41467-021-22177-1 (PMC8009873; doi:10.1038/s41467-021-22177-1)
Supplement: Supplementary file 1 — Supplementary Information [file 41467_2021_22177_MOESM1_ESM.pdf]

Supplementary Information

Table of Contents

SUPPLEMENTARY TABLE 1. EXPLORATORY EFFECT MODIFIER RESULTS FOR PRIMARY OUTCOME (VIRAL CESSATION) 2

SUPPLEMENTARY FIGURE 1. SYMPTOM DURATION AT PRESENTATION 3

SUPPLEMENTARY FIGURE 2. INDIVIDUAL PARTICIPANT SYMPTOMS OVER COURSE OF STUDY, STRATIFIED BY SYMPTOM COMPLEX AND TREATMENT ARM. 4

SUPPLEMENTARY FIGURE 3. OXYGEN SATURATIONS BETWEEN OVER TIME, STRATIFIED BY TREATMENT ARM 5

SUPPLEMENTARY FIGURE 4. OROPHARYNGEAL VIRAL LOAD OVER TIME 6

SUPPLEMENTARY FIGURE 5. TIME UNTIL SARS-COV-2 VIRAL SHEDDING CESSATION FROM OROPHARYNGEAL SWABS STRATIFIED BY BASELINE SARS-COV-2 IGG SEROPOSITIVITY 7

SUPPLEMENTARY FIGURE 6. TIME UNTIL SARS-COV-2 VIRAL SHEDDING CESSATION FROM OROPHARYNGEAL SWABS STRATIFIED BY BASELINE OROPHARYNGEAL VIRAL LOAD 8

SUPPLEMENTARY FIGURE 7. ALANINE TRANSAMINASE LEVELS MEASURED LONGITUDINALLY BY TREATMENT ARM 9

APPENDIX 1: DAILY SYMPTOM QUESTIONNAIRE 10

Supplementary Table 1. Exploratory Effect Modifier Results for Primary Outcome (Viral Cessation)

Effect modifier	Interaction p-value	Subgroup	Median time (95% CI) to shedding cessation		Hazard ratio (95% CI) for lambda vs placebo
			Lambda	Placebo	
Ct value < 30	0.15	Ct < 30	15 (14, undefined)	11 (10, 14)	0.51 (0.26-1.04)
		Ct 30+	3 (3, 7)	5 (2, 7)	0.95 (0.60, 1.52)
Seropositivity	0.03	Seronegative	13 (7, 21)	10 (7, 13)	0.66 (0.39, 1.10)
		Seropositive	1 (0, 7)	5 (2, 10)	1.58 (0.88, 2.86)
Risk score	0.77	NA	NA	NA	1.05 (0.76, 1.45)
Age 50+	0.21	Age < 50	7 (5, 14)	7 (5, 10)	1.20 (0.59, 2.47)
		Age 50+	3 (3, 14)	7 (5, 21)	0.70 (0.44, 1.10)
Male	0.74	Female	7 (3, 14)	5 (5, 12)	0.86 (0.52, 1.42)
		Male	7 (5, 14)	10 (7, 11)	0.75 (0.42, 1.36)

CT: Cycle Threshold by real time quantit:
Risk score is defined as the number of relevant severe disease risk factors present at baseline (presence of either temperature of 99.5F+, cough, or shortness of breath; age 60+; male sex; Black race; Hispanic ethnicity; BMI 30+; ALC<1000; ALT 94+).
Hazard ratio >1 favors faster shedding cessation in Lambda vs. placebo arms; hazard ratio <1 favors delayed shedding cessation in Lambda vs. placebo arms. All models adjusted for age group and sex.

Supplementary Figure 1. Symptom duration at presentation

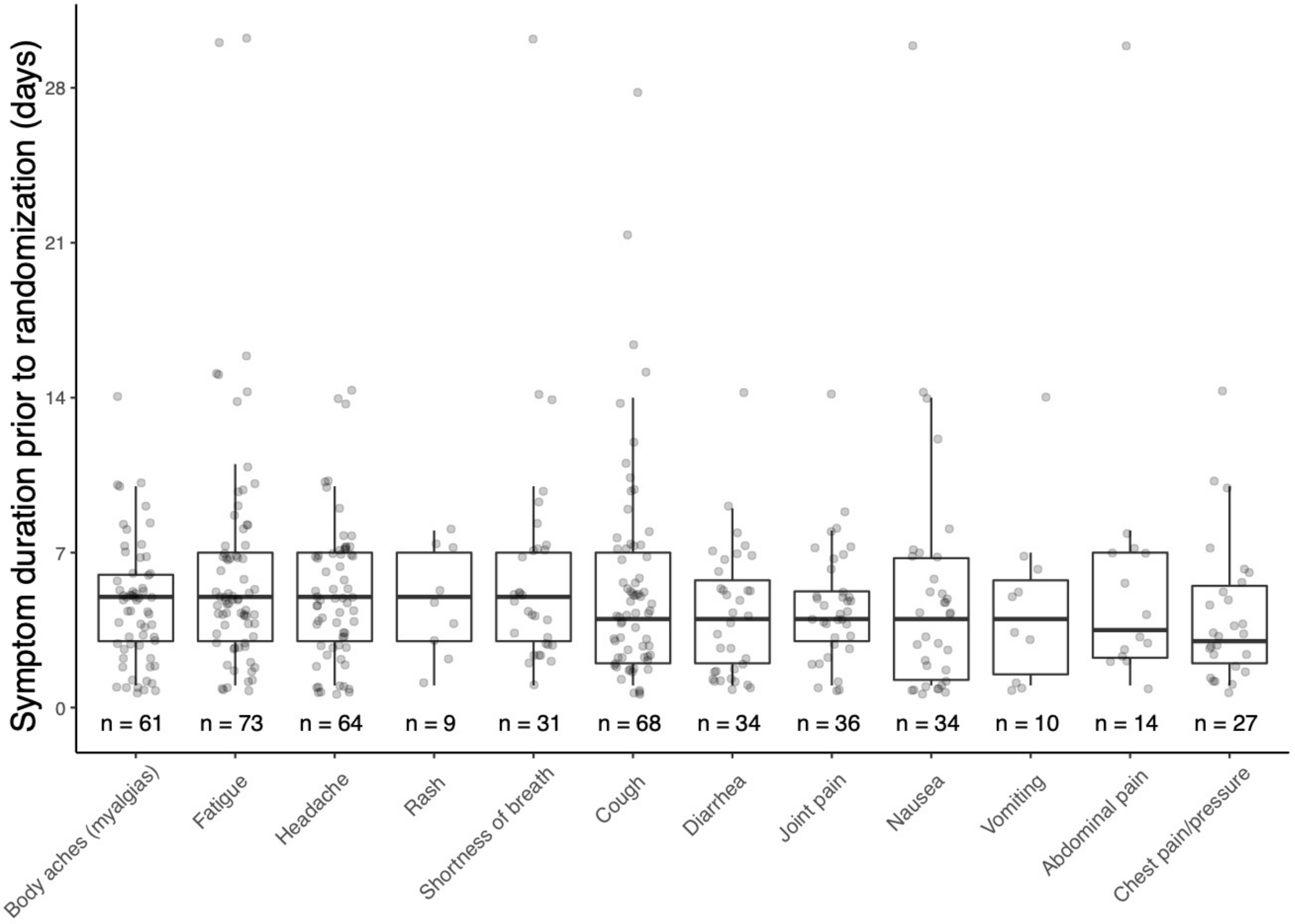

Supplementary Figure 1. Symptom duration at presentation. Points show symptom duration for each individual. Boxplots show median symptom duration, and upper and lower hinges correspond to first (25%) and third (75%) quartiles.

Supplementary Figure 2. Individual participant symptoms over course of study, stratified by symptom complex and treatment arm.

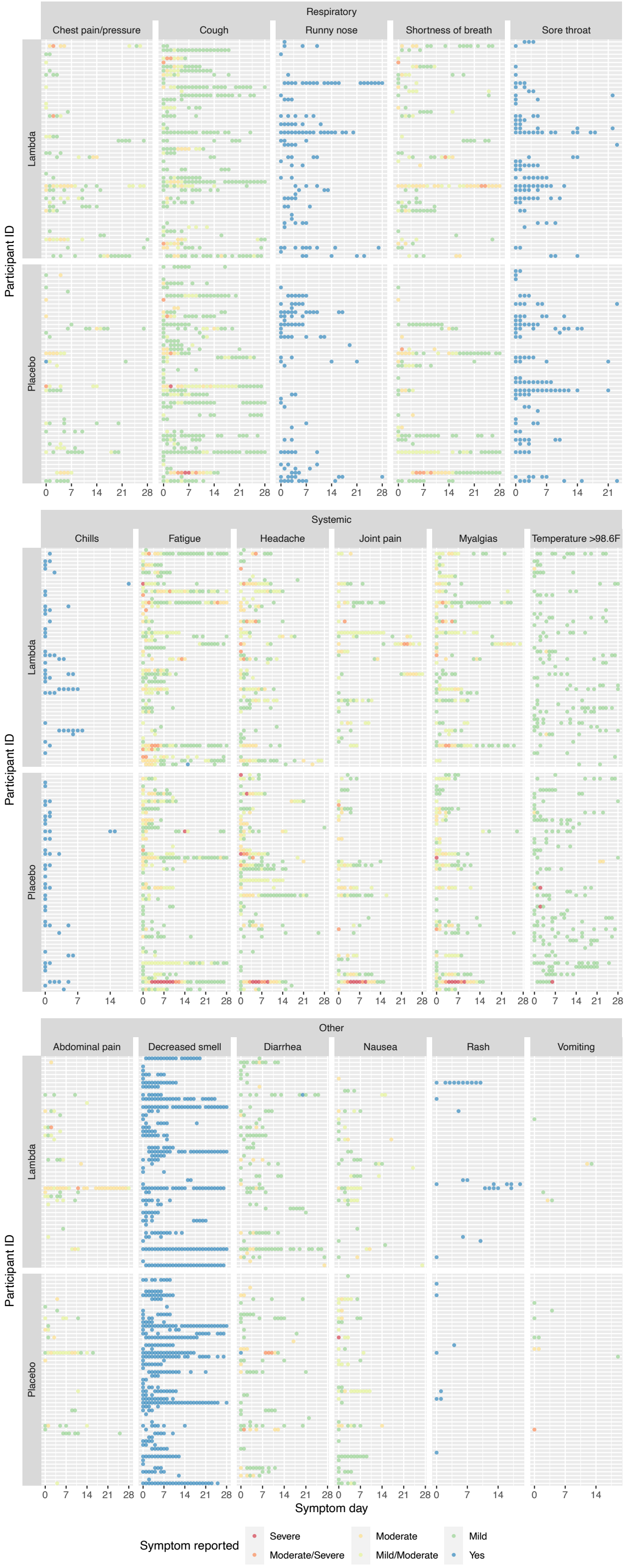

Supplementary Figure 2. Individual participant symptoms, stratified by symptom complex (respiratory, systemic, or other) and treatment arm. Colors represent severity of symptom reported, or symptom presence (blue circles) for those symptoms where severity was not assessed.

Supplementary Figure 3. Oxygen saturations between over time, stratified by treatment arm

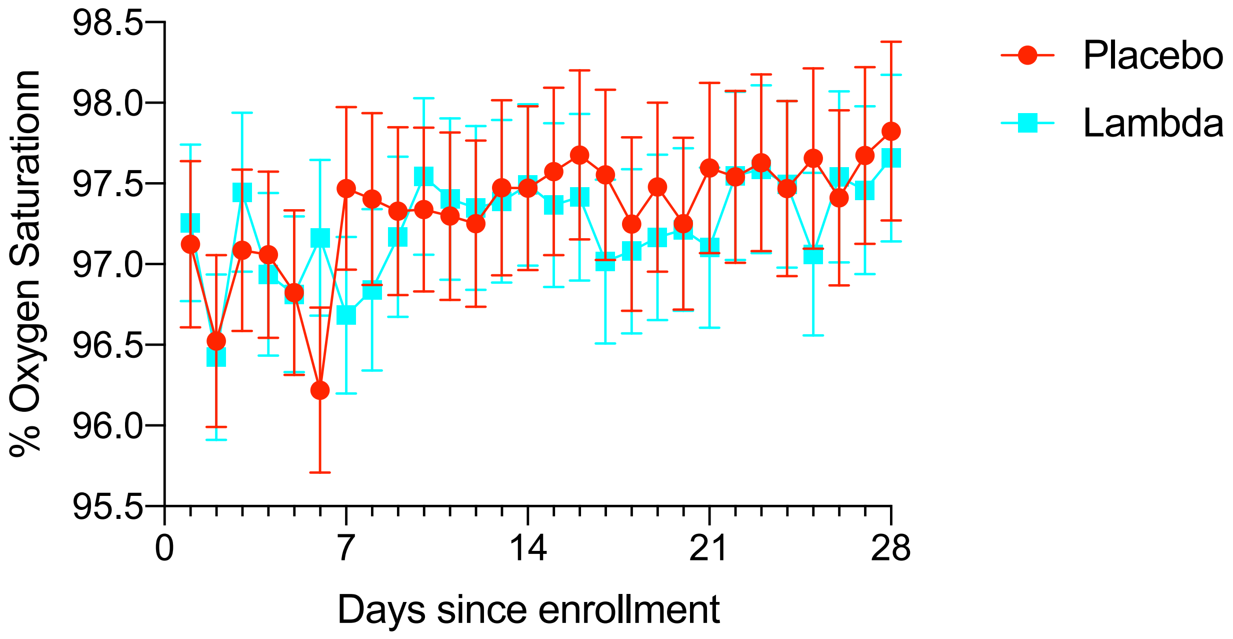

Supplementary Figure 3. At-home oxygen saturation measurements over course of study, stratified by treatment arm. Patients given oxygen saturation monitors and self-reported oxygen levels on daily symptom surveys. Point estimates quantified by linear models using generalized estimating equations with robust standard errors accounting for repeated measures per participant. Each point represents marginal estimates of mean daily oxygen saturation from 40-60 individuals with available data per study arm with 95% CI, stratified by treatment arm. Oxygen saturation did not differ significantly between arms (Coef -0.09, 95% CI -0.49-0.31, P=0.66).

Supplementary Figure 4. Oropharyngeal viral load over time

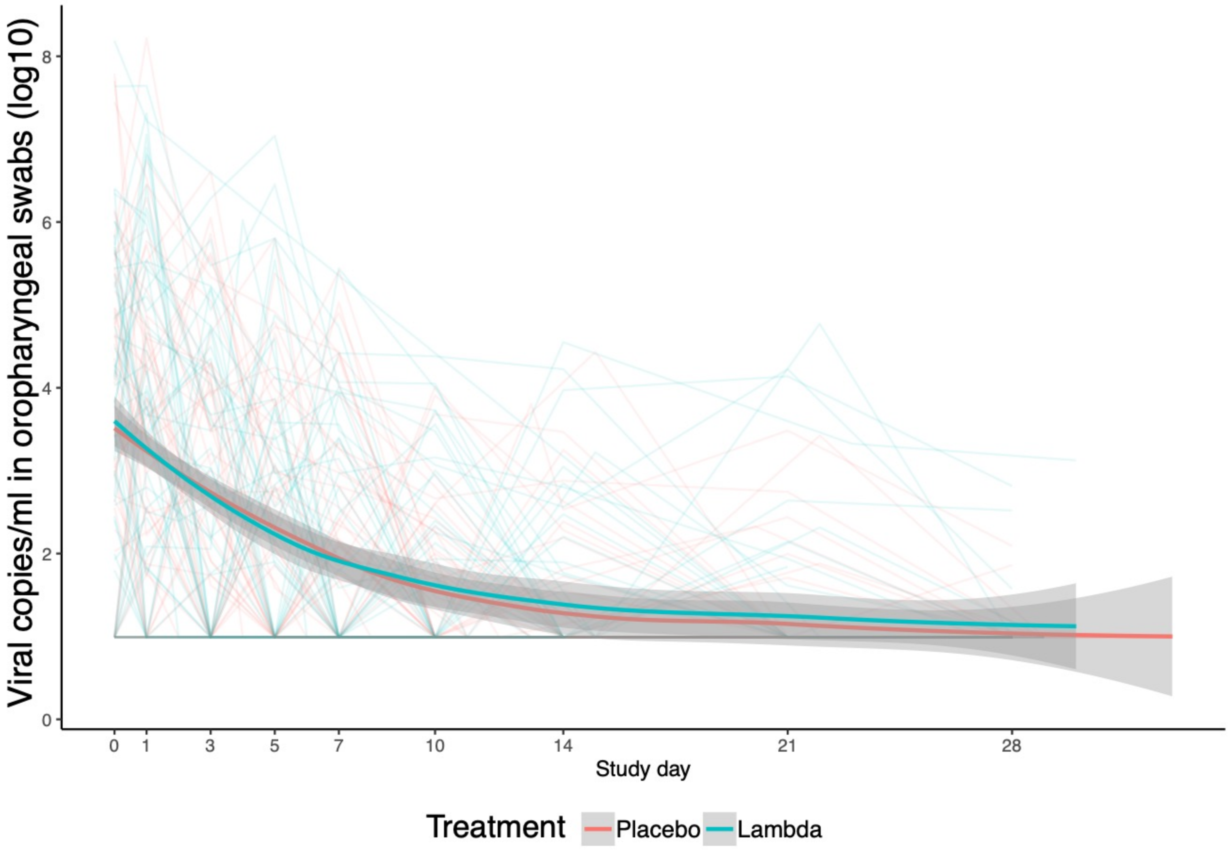

Supplementary Figure 4. Viral copies/ml in oropharyngeal swabs over course of study, by participant and treatment arm. Shown are LOESS regression lines depicting median viral copies/ml (solid lines) with 95% CI (shaded areas), stratified by treatment arm.

Supplementary Figure 5. Time until SARS-CoV-2 viral shedding cessation from oropharyngeal swabs stratified by baseline SARS-CoV-2 IgG seropositivity

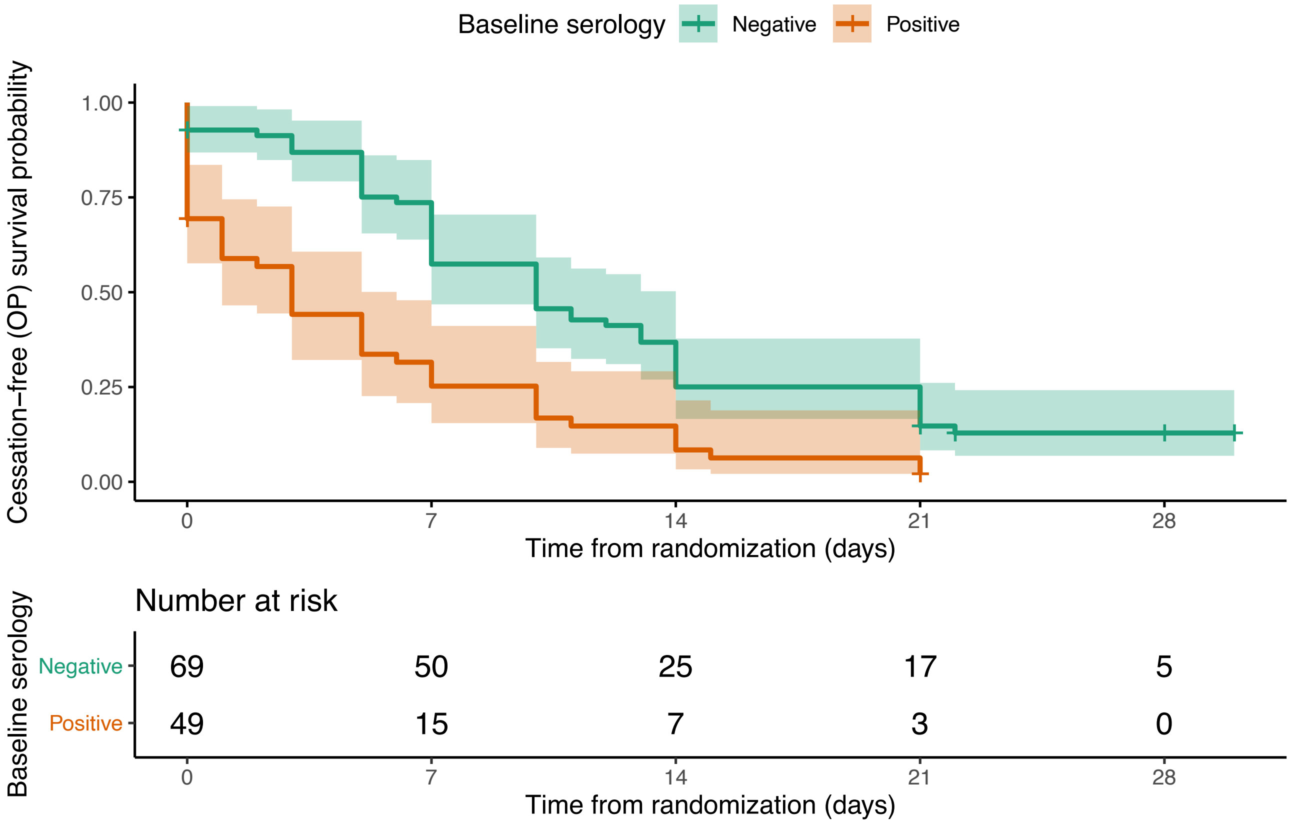

Supplementary Figure 5. Kaplan-Meier Analyses of the time until cessation of SARS-CoV-2 viral shedding from oropharyngeal swabs stratified by baseline SARS-CoV-2 seropositivity, Seronegative (green) vs. Seropositive (red). Solid lines represent Kaplan-Meier survival probability; shading represents 95% confidence intervals.

Supplementary Figure 6. Time until SARS-CoV-2 viral shedding cessation from oropharyngeal swabs stratified by baseline oropharyngeal viral load

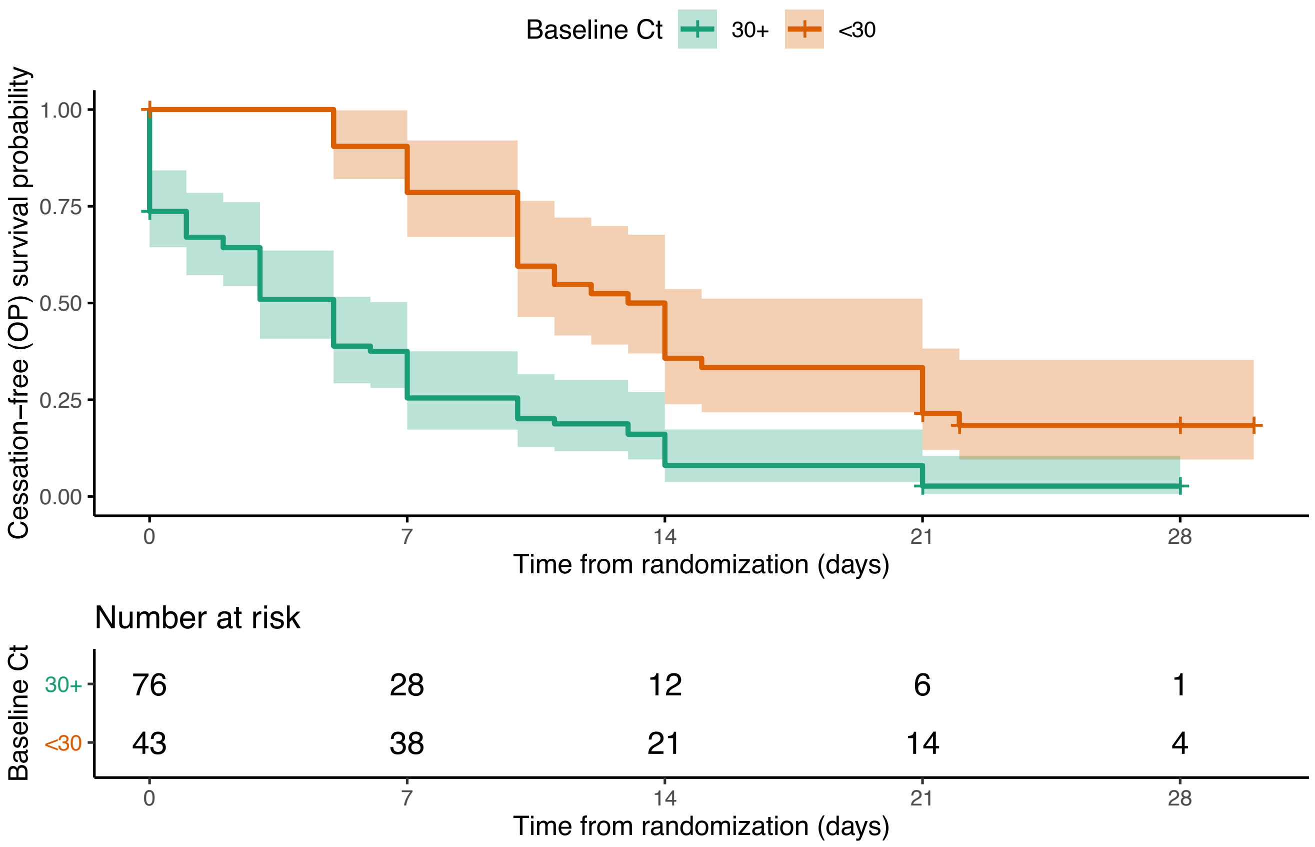

Supplementary Figure 6. Kaplan-Meier Analyses of the time until cessation of SARS-CoV-2 viral shedding from oropharyngeal swabs stratified by baseline oropharyngeal SARS-CoV-2 cycle threshold, >30(green) vs. <30 (red). Solid lines represent Kaplan-Meier survival probability; shading represents 95% confidence intervals.

Supplementary Figure 7. Alanine transaminase levels measured longitudinally by treatment arm

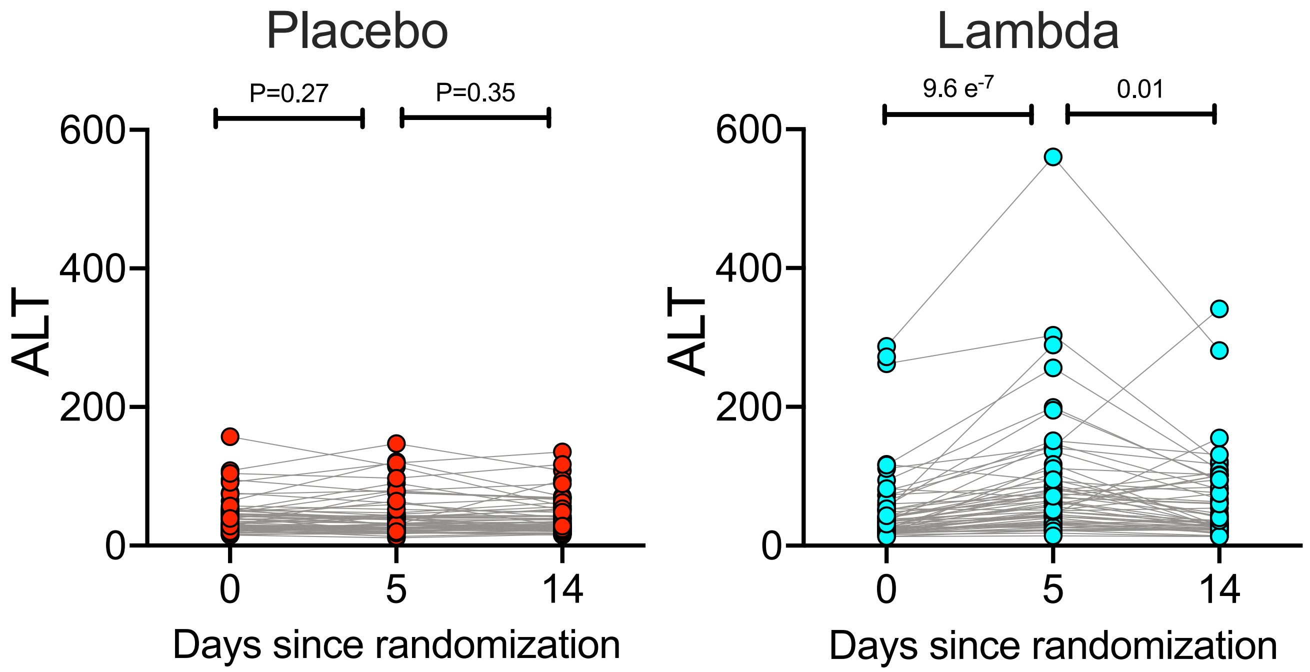

Supplementary Figure 7. Alanine transaminase levels measured at day 0 and day 5 and 14 post-randomization in Placebo (red) and Lambda (blue) arms. Measurements compared within individuals and between timepoints using paired, two-tailed t-tests, without adjustment for multiple comparisons.

Appendix 1: Daily symptom questionnaire

Daily Record Of Symptoms

SECTION

Please take your oral temperature and assess your oxygen saturation with your pulse oximeter. *Por favor tome su temperatura oral y mida su saturación oxigeno con el oximetro*

____

Oral temperature. *Temperatura oral* ______________________________

Time temperature was obtained. *Hora en que la temperatura se obtuvo* ______________________________

Blood oxygen saturation *Saturación de oxigeno sanguineo* ______________________________

Did you take a nasal swab today? *Se tomo un exudado nasal el dia de hoy?*

- ◯ Yes
- ◯ No

If it is written on your schedule (clinic visit day 3, 14, 28): did you collect your stool sample for your next visit? *Levanto su muestra de heces para su proxima visita?*

- ◯ Yes, I have collected my sample
- ◯ No, I am scheduled and did not collect my sample
- ◯ No, I am NOT scheduled to collect my sample

For the following questions, please consider any symptoms you are currently experiencing, or have been experiencing, since completing your last survey. *Para las siguientes preguntas, por favor considere* ***cualquier sintoma que haya tenido? experimentado?*** *desde que contesto la ultima encuesta.*

____

Do you have a cough? *Ha tenido tos?*

- ◯ Yes
- ◯ No

How bad is your cough? *Que tan mal es su tos?*

- ◯ Mild; just a few coughs per day Leve, pocos tosidos por dia
- ◯ Mild/Moderate / Leve/Moderado
- ◯ Moderate; frequent but I can tolerate it Frequente pero lo tolero
- ◯ Moderate/Severe / Moderado/Severo
- ◯ Severe; I am very uncomfortable Severo; estoy muy incomodo

Are you short of breath? *Tiene dificultad para respirar?*

- ◯ Yes
- ◯ No

How short of breath are you? *Como es la dificultad de respirar?*

- ◯ Mild; just short of breath with exercise FaltaAlientoEjercicio
- ◯ Mild/Moderate / Leve/Moderado
- ◯ Moderate; I get short of breath doing daily activities FaltaAlientoActivDiaria
- ◯ Moderate/Severe / Moderado/Severo
- ◯ Severe; I feel I can't get enough air even at rest Severo;FaltaAireReposo

Are you more fatigued than normal? *Esta mas fatigado de lo normal?*

- ◯ Yes
- ◯ No

How fatigued are you? *Que tan fatigado/a esta?*

- ◯ Mild; I go about my day normally Leve; puedo hacer mi dia normal
- ◯ Mild/Mod Leve/Moderado
- ◯ Moderate; I rest more and restrict activity Moderado; DescansoPocaActivi
- ◯ Mod/Sev Moderado/Severo
- ◯ Severe; I am staying in bed I'm so tired Severo; EnCamaMuyCansado

Do you have a sore throat? *Tiene la garganta irritada?*

- ◯ Yes
- ◯ No

Do you have any chills? *Tiene escalofrios?*

- ◯ Yes
- ◯ No

Do you have a runny nose? *Tiene nariz floja o goteo?*

- ◯ Yes
- ◯ No

Is your sense of taste or smell decreased from normal?
Han disminuido de lo normal su sentido del olfato o gusto?

- ◯ Yes
- ◯ No

Do you have a headache? *Tiene dolor de cabeza?*

- ◯ Yes
- ◯ No

How bad is your headache? *Que tan mal es su dolor de cabeza?*

- ◯ Mild; I can ignore it Leve; puedo ignorarle
- ◯ Mild/Moderate / Leve/Moderado
- ◯ Moderate; I need to take medication Moderado; NecesitoMedicamento
- ◯ Moderate/Severe Moderado/Severo
- ◯ Severe; it is markedly limiting my life Severo; LimitaMiVida

Do you have body aches (myalgias)? *Tiene dolor de cuerpo (mialgias)?*

- ◯ Yes
- ◯ No

How bad are your body aches? *Que tan mal es su dolor de cuerpo?*

- ◯ Mild; I can ignore them Leve; puedo ignorarle
- ◯ Mild/Moderate Leve/Moderado
- ◯ Moderate; I need to restrict some activities Moderado; LimitaActividades
- ◯ Moderate/Severe Moderado/Severo
- ◯ Severe; they are markedly limiting my life Severo; LimitaMiVida

Do you have any pain in your joints? *Tiene dolor en sus articulaciones?*

- ◯ Yes
- ◯ No

How bad are your joint aches? *Que tan mal son sus dolores de articulaciones?*

- ◯ Mild; I can ignore them Leve; puedo ignorarle
- ◯ Mild/Moderate Leve/Moderado
- ◯ Moderate; I need to restrict some activities Moderado; LimitaActividades
- ◯ Moderate/Severe Moderado/Severo
- ◯ Severe; they are markedly limiting my life Severo/LimitaMiVida

Do you have any pain or pressure in your chest? *Tiene dolor o presion en el pecho?*

- ◯ Yes
- ◯ No

How bad is your chest pain/pressure? *Que tan mal es su dolor /presion en el pecho?*

- ◯ Mild; I feel it occasionally but can ignore it most of the time Leve; Ignoro
- ◯ Mild/Mod Leve/Moderado
- ◯ Moderate; I notice it al lot and it limits my activity Moderado; LimitaActivid
- ◯ Mod/Sev Moderado/Severo
- ◯ Severe; I have bad pain and pressure that bothers me most of the time Severo; dolor y presion molesta todo tiempo

Do you have any nausea? *Tiene nausea?*

- ◯ Yes
- ◯ No

How bad is your nausea? *Que tan mal es su nausea?*

- ◯ Mild; I'm eating and ignoring it Leve; Estoy comiendo y le ignoro
- ◯ Mild/Mod Leve/Moderada
- ◯ Moderate; I don't want to eat and can't ignore it Mod;NoComoNoIgnoro
- ◯ Mod/Sev Moderada, Severa
- ◯ Severe; I am feeling quite uncomfortable Severa, SIentoMuyIncomodo/a

Have you vomited? *Ha vomitado?*

- ◯ Yes
- ◯ No

How many times have you vomited today? *Cuantas veces vomito hoy?*

____

Do you have any abdominal pain? *Tiene algun dolor abdominal?*

- ◯ Yes
- ◯ No

How bad is your abdominal pain? *Que tan mal es su dolor abdominal?*

- ◯ Mild; I can ignore it Leve; puedo ignorarle
- ◯ Mild/Moderate Leve/Moderado
- ◯ Moderate; it is limiting my activities Moderado; LimitaActividades
- ◯ Moderate/Severe Moderado/Severo
- ◯ Severe; it hurts a lot. I may need to see a doctor Severo; duele, NecesitoDr

Have you had any diarrhea? (three or more loose or watery stools in 24 hours) *Ha tenido diarrea? (tres o mos veces evacuaciones flojas o liquidas en 24 horas)*

- ◯ Yes
- ◯ No

Number of times you had loose or watery stool within the last 24 hours?
Numero de veces que ha tenido diarrea floja o liquida en las ultimas 24 horas?

- ◯ Less than 3 times Menos de 3 veces
- ◯ Between 3 and 6 times Entre 3 y 6 veces
- ◯ More than 6 times Mas de 6 veces

Are you having pain at the site of the injection? *Ha tenido dolor en el sitio de la inyección?*

- ◯ Yes
- ◯ No

Is this pain limiting your activity? *El dolor le limita su actividad?*

- ◯ Yes
- ◯ No

Do you have a new rash? *Tiene alguna erupcion nueva?*

- ◯ Yes
- ◯ No

My rash is: (check all the apply) *Su erupción es: (Marque todas las que apliquen)*

- ☐ Only around the injection site Solo alrededor de la inyección
- ☐ Covering a small amount of my body CubrePartePequeñaDelCuerpo
- ☐ Extensive covering much of my body ExtensaCubreCasiMiCuerpo
- ☐ Involving the inside of my mouth or lips EstaDentroBocaOLabios
- ☐ Itchy Comezon
- ☐ Itchy with hives Comezon con ronchas /manchas

Do you have any other symptoms you would like to tell us about? *Tiene otro sintoma que le gustaria mencionar?*

- ○ Yes
- ○ No

Please tell us about these symptoms. *Por favor describa estos sintomas?*

____

IF YOUR SYMPTOMS NEED URGENT ATTENTION, CALL 911. IF YOU ARE CONCERNED BUT YOU DON'T THINK YOUR SYMPTOMS ARE URGENT, CALL THE STUDY OFFICE AT 650-721-9316.
SI SUS SINTOMAS SON URGENTES, LLAME AL 911. SI USTED QUIERE HABLAR CON ALGUIEN Y SUS SINTOMAS NO SON URGENTES, 650 721-9316

____

Is there anything you think it is important for us to know? *Hay algo mas que es importatnte y debemos saber?*

- ○ Yes
- ○ No

Use this space to tell us what you would like us to know. *Use el espacio para decirnos lo que quiere que dejarnos saber.*

____
