# Supplementary material for: Peginterferon Lambda-1a for treatment of outpatients with uncomplicated COVID-19: a randomized placebo-controlled trial
Source: Nat Commun. 2021 Mar 30;12:1967. doi: 10.1038/s41467-021-22177-1 (PMC8009873; doi:10.1038/s41467-021-22177-1)
Supplement: Supplementary file 3 — Reporting Summary [file 41467_2021_22177_MOESM3_ESM.pdf]

nature research

Corresponding author(s): Prasanna Jagannathan

Last updated by author(s): Nov 17, 2020

Reporting Summary

Nature Research wishes to improve the reproducibility of the work that we publish. This form provides structure for consistency and transparency in reporting. For further information on Nature Research policies, see our Editorial Policies and the Editorial Policy Checklist.

Statistics

For all statistical analyses, confirm that the following items are present in the figure legend, table legend, main text, or Methods section.

n/a	Confirmed	
☐	☒	The exact sample size (*n*) for each experimental group/condition, given as a discrete number and unit of measurement
☐	☒	A statement on whether measurements were taken from distinct samples or whether the same sample was measured repeatedly
☐	☒	The statistical test(s) used AND whether they are one- or two-sided<br>*Only common tests should be described solely by name; describe more complex techniques in the Methods section.*
☐	☒	A description of all covariates tested
☐	☒	A description of any assumptions or corrections, such as tests of normality and adjustment for multiple comparisons
☐	☒	A full description of the statistical parameters including central tendency (e.g. means) or other basic estimates (e.g. regression coefficient) AND variation (e.g. standard deviation) or associated estimates of uncertainty (e.g. confidence intervals)
☐	☒	For null hypothesis testing, the test statistic (e.g. *F*, *t*, *r*) with confidence intervals, effect sizes, degrees of freedom and *P* value noted<br>*Give P values as exact values whenever suitable.*
☒	☐	For Bayesian analysis, information on the choice of priors and Markov chain Monte Carlo settings
☒	☐	For hierarchical and complex designs, identification of the appropriate level for tests and full reporting of outcomes
☐	☒	Estimates of effect sizes (e.g. Cohen's *d*, Pearson's *r*), indicating how they were calculated

Our web collection on statistics for biologists contains articles on many of the points above.

Software and code

Policy information about availability of computer code

Data collection	Redcap Cloud Version 1.5, Redcap Academic version 9.1.0
Data analysis	R Version 4.0.2

For manuscripts utilizing custom algorithms or software that are central to the research but not yet described in published literature, software must be made available to editors and reviewers. We strongly encourage code deposition in a community repository (e.g. GitHub). See the Nature Research guidelines for submitting code & software for further information.

Data

Policy information about availability of data

All manuscripts must include a data availability statement. This statement should provide the following information, where applicable:

- Accession codes, unique identifiers, or web links for publicly available datasets
- A list of figures that have associated raw data
- A description of any restrictions on data availability

The datasets generated and analyzed during the current study are available in the Stanford Digital Repository. Available at: https://purl.stanford.edu/hc972ys6733

Field-specific reporting

Life sciences study design

All studies must disclose on these points even when the disclosure is negative.

Sample size	Assuming 1:1 randomization and the use of a two-sided log rank test at the alpha=0.04999 level of significance for the final analysis, we anticipated the occurrence of 79 shedding cessation events, which provided 80% power to detect a hazard ratio of 2.03. We additionally assumed median time to shedding cessation of 14 days in the control arm and 7 days in the treatment arm, a two-month accrual period, a two-week follow-up period after randomization of the last patient, and 10% drop out in the control arm. This enabled an interim analysis conducted at alpha=0·00001 to assess overwhelming efficacy after 50% of participants completed 24 hours of follow-up. We estimated that the total sample size required to achieve 79 events was 120 (60 participants per arm).
Data exclusions	No data were excluded from the analysis
Replication	This was a single-site, randomized clinical trial replicated in 60 independent participants per arm (120 total). All individuals were included in the final analysis.
Randomization	120 eligible patients were randomly assigned to Lambda or placebo in a 1:1 ratio to the treatment and control arms using a REDCAP-based computer-generated randomization scheme developed by the study data management provider. Randomization was stratified by age (>=50 and <50 years old) and sex. The study data management provider completed a password-protected electronic spreadsheet containing the randomization allocation, along with the code used to generate the allocation and the seed used in the random number generation. These are stored on secure servers at Stanford.
Blinding	The study medication/placebo syringe was dispensed by the Stanford Investigational Pharmacist and administered by a study nurse. Lambda and placebo syringes were identically labeled but differed in the appearance of the needle hub. Since the nurse administering the medication might see syringe differences, the study was not strictly “double-blind” even though all participants and investigators were blinded to treatment arm.

Reporting for specific materials, systems and methods

We require information from authors about some types of materials, experimental systems and methods used in many studies. Here, indicate whether each material, system or method listed is relevant to your study. If you are not sure if a list item applies to your research, read the appropriate section before selecting a response.

Materials & experimental systems

n/a	Involved in the study	
☒	☐	Antibodies
☒	☐	Eukaryotic cell lines
☒	☐	Palaeontology and archaeology
☒	☐	Animals and other organisms
☐	☒	Human research participants
☐	☒	Clinical data
☒	☐	Dual use research of concern

Methods

n/a	Involved in the study	
☒	☐	ChIP-seq
☒	☐	Flow cytometry
☒	☐	MRI-based neuroimaging

Human research participants

Policy information about studies involving human research participants

Population characteristics	We enrolled 120 participants, of whom 110 (91.7%) completed 28 days of follow up (Fig 1a). The median age was 36 years (range 18-71), 50 participants (41.7%) were female, and 75 (62.5%) were Latinx ethnicity (Table 1). Eight (6.7%) participants were asymptomatic at baseline. Of those with symptoms, the median duration of symptoms prior to randomization was five days.
Recruitment	Adults aged 18-65 years with an FDA emergency use authorized reverse transcription-polymerase chain reaction (RT-PCR) positive for SARS-CoV-2 within 72 hours from swab to the time of enrolment were eligible for participation in this study. Participants were recruited with flyers, online advertising, and phone calls to Stanford patients with positive SARS-CoV-2 RT-PCR. Recruitment materials and phone calls were provided in multiple languages, including English and Spanish. After confirming eligibility and providing informed consent in the patient's primary language, participants underwent a standardized history and physical exam, and completed bloodwork. If inclusion criteria were met, participants were randomly assigned to Lambda or placebo. Inclusion of asymptomatic patients (<10% of study) may have slightly biased study results, since these individuals present with lower viral RNA levels and could not contribute to analyses of symptom alleviation. However, both symptomatic and asymptomatic patients were included given reports that presence of infectious virus were similar in samples from asymptomatic and symptomatic persons. As recruitment was performed via convenience sampling, study findings may not be generalizable to other settings. Importantly, we prioritized recruitment of LatinX participants the majority (62.5%) of participants in our study were Latinx, reflecting the high burden of COVID-19 among the Latinx community in our surrounding communities. However, this may bias our results
Ethics oversight	The study was performed as an investigator initiated clinical trial with the FDA (IND 419217), and approved by the Institutional Review Board of Stanford University.

Note that full information on the approval of the study protocol must also be provided in the manuscript.

Clinical data

Policy information about clinical studies

All manuscripts should comply with the ICMJEguidelines for publication of clinical research and a completedCONSORT checklist must be included with all submissions.

Clinical trial registration	ClinicalTrials.Gov, NCT04331899; FDA, IND 419217
Study protocol	Full study protocol provided with the submission, and published in the Stanford Digital Repository (https://purl.stanford.edu/hc972ys6733)
Data collection	The trial was conducted within the Stanford Health Care System. We enrolled 120 participants between April 25 and July 17, 2020. Participants were followed for 28 days.
Outcomes	The primary outcome was time to first of two consecutive negative oropharyngeal tests for SARS-CoV-2 by RT-PCR. Secondary outcomes included: 1) Time to alleviation of all symptoms, defined as time until the first day when no symptoms were reported; 2) SARS-CoV-2 oropharyngeal viral load over time; 3) SARS-CoV-2 viral load area under the curve (AUC); and 4) Incidence of emergency department visits or hospitalizations within 28 days of initiation of treatment. Adverse events (AEs) and serious AEs (SAEs) were the primary safety endpoints. Exploratory outcomes included: 1) Time until sustained resolution of symptoms, defined as the first day when no symptoms were reported for the duration of the study; 2) Progression of disease, defined as admission to the emergency department, hospitalization, or worsening cough or shortness of breath defined as an increase in severity of two points or more on a five-point scale. All outcomes and analyses were pre-specified in a statistical analysis plan prior to database lock and unblinding.
