# Supplementary material for: Peginterferon Lambda-1a for treatment of outpatients with uncomplicated COVID-19: a randomized placebo-controlled trial
Source: Nat Commun. 2021 Mar 30;12:1967. doi: 10.1038/s41467-021-22177-1 (PMC8009873; doi:10.1038/s41467-021-22177-1)
Supplement: Supplementary file 2 — Peer Review File [file 41467_2021_22177_MOESM2_ESM.pdf]

Editorial Note: This manuscript has been previously reviewed at another journal that is not operating a transparent peer review scheme. This document only contains reviewer comments and rebuttal letters for versions considered at *Nature Communications.*

REVIEWERS' COMMENTS

Reviewer #1 (Remarks to the Author):

I have reviewed the response to reviewers. I appreciate the detailed response. They have addressed each point, and in some cases significantly added to the manuscript.

I re-read the results and discussion. These section seem much clearer in conveying results and more balanced in tone. Fundamentally, this manuscript describes a well conducted study and in a field that is of great interest. While the results were negative, they are important to convey. I have no lingering concerns with the manuscript.

Point by Point Response to Reviewers

<u>Reviewer #1:</u>

I have reviewed the response to reviewers. I appreciate the detailed response. They have addressed each point, and in some cases significantly added to the manuscript.

I re-read the results and discussion. These section seem much clearer in conveying results and more balanced in tone. Fundamentally, this manuscript describes a well conducted study and in a field that is of great interest. While the results were negative, they are important to convey. I have no lingering concerns with the manuscript.

Response: We thank the reviewer for their review of this manuscript.
